# Supplementary material for: The interactome of intact mitochondria by cross-linking mass spectrometry provides evidence for coexisting respiratory supercomplexes
Source: Mol Cell Proteomics. 2017 Dec 8;17(2):216–32. doi: 10.1074/mcp.RA117.000470 (PMC5795388; doi:10.1074/mcp.RA117.000470)
Supplement: Supplemental Data [file supp_RA117.000470_133922_0_supp_23978_fzffwf.zip › spectra_annotation/mito_DR_spectra_annotation/261-1-4-1-5-1.pdf]

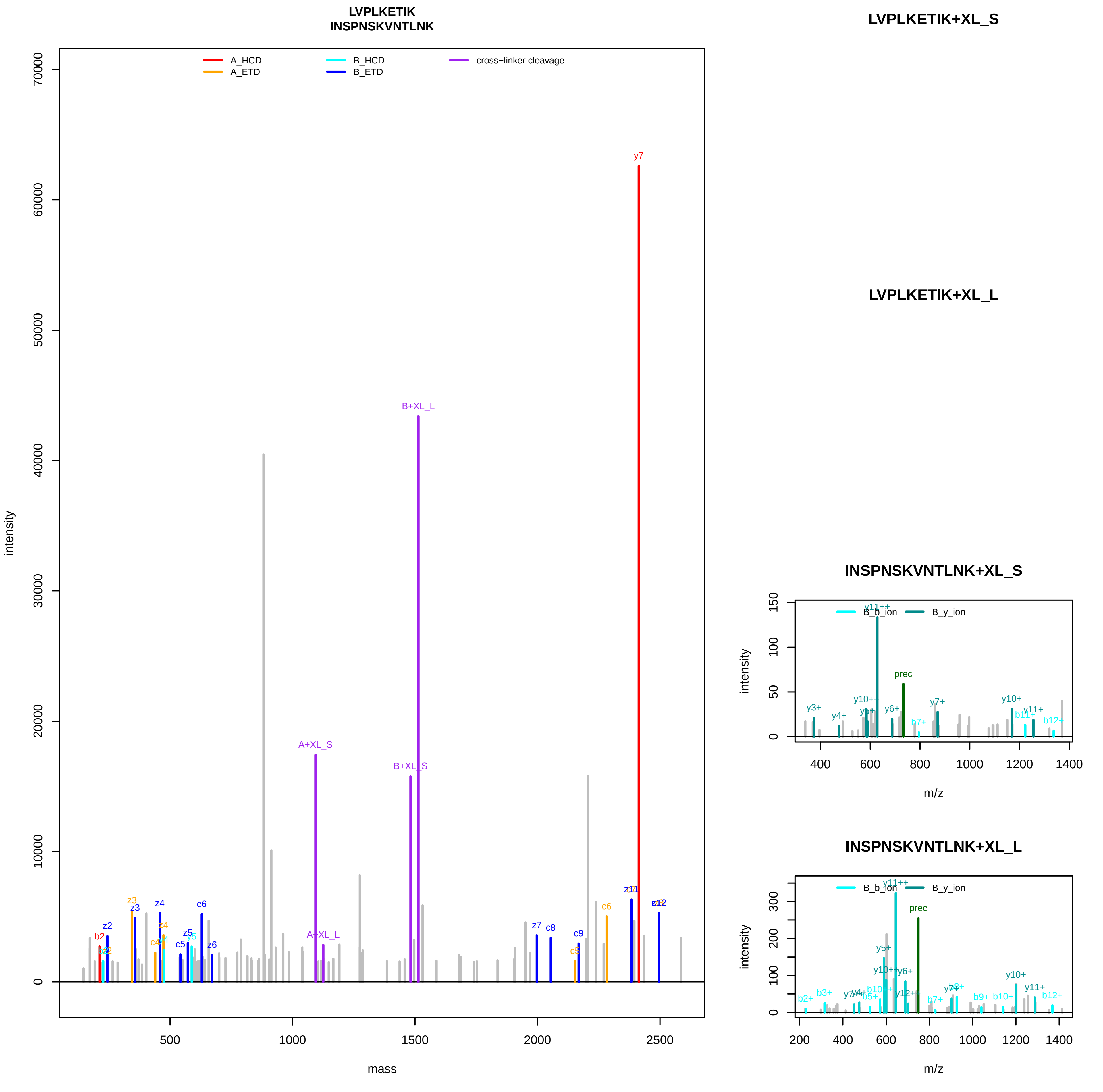
LVPLKETIK
INSPNSKVNTLNK
A_HCD
A_ETD
B_HCD
B_ETD
cross−linker cleavage
intensity
mass
y7
B+XL_L
A+XL_S
B+XL_S
A+XL_L
b2
z2
z3
z4
c4
z5
y5
c5
c6
z6
z7
c8
c9
c5
c6
z11
z12
LVPLKETIK+XL_S
LVPLKETIK+XL_L
INSPNSKVNTLNK+XL_S
B_b_ion
B_y_ion
y11++
prec
y3+
y4+
y10++
y5+
y6+
b7+
y7+
y10+
b11+
y11+
b12+
m/z
INSPNSKVNTLNK+XL_L
b2+
b3+
b5+
b10++
y12++
b8+
b9+
b10+
